# Spinoparabrachial projection neurons form distinct classes in the mouse dorsal horn

**DOI:** 10.1097/j.pain.0000000000002194

**Published:** 2021-02-22

**Authors:** Tyler J. Browne, Kelly M. Smith, Mark A. Gradwell, Jacqueline A. Iredale, Christopher V. Dayas, Robert J. Callister, David I. Hughes, Brett A. Graham

**Affiliations:** a School of Biomedical Sciences and Pharmacy, Faculty of Health, University of Newcastle, Callaghan, New South Wales, Australia; b Hunter Medical Research Institute (HMRI), New Lambton Heights, New South Wales, Australia; c Department of Neurobiology and the Pittsburgh Center for Pain Research, University of Pittsburgh, Pittsburgh, PA, United States; d Department of Cell Biology and Neuroscience, Rutgers, The State University of New Jersey, Piscataway, NJ, United States; e W.M. Keck Center for Collaborative Neuroscience, Rutgers, The State University of New Jersey, Piscataway, NJ, United States; f Institute of Neuroscience Psychology, College of Medical, Veterinary and Life Sciences, University of Glasgow, Glasgow, United Kingdom

**Keywords:** Patch clamp, Electrophysiology, Spinal cord, Morphology, Pain

## Abstract

Projection neurons in the spinal dorsal horn relay sensory information to higher brain centres. The activation of these populations is shaped by afferent input from the periphery, descending input from the brain, and input from local interneuron circuits. Much of our recent understanding of dorsal horn circuitry comes from studies in transgenic mice; however, information on projection neurons is still based largely on studies in monkey, cat, and rat. We used viral labelling to identify and record from mouse parabrachial nucleus (PBN) projecting neurons located in the dorsal horn of spinal cord slices. Overall, mouse lamina I spinoparabrachial projection neurons (SPBNs) exhibit many electrophysiological and morphological features that overlap with rat. Unbiased cluster analysis distinguished 4 distinct subpopulations of lamina I SPBNs, based on their electrophysiological properties that may underlie different sensory signalling features in each group. We also provide novel information on SPBNs in the deeper lamina (III-V), which have not been previously studied by patch clamp analysis. These neurons exhibited higher action potential discharge frequencies and received weaker excitatory synaptic input than lamina I SPBNs, suggesting this deeper population produces different sensory codes destined for the PBN. Mouse SPBNs from both regions (laminae I and III-V) were often seen to give off local axon collaterals, and we provide neuroanatomical evidence they contribute to excitatory input to dorsal horn circuits. These data provide novel information to implicate excitatory input from parabrachial projection neuron in dorsal horn circuit activity during processing of nociceptive information, as well as defining deep dorsal horn projection neurons that provide an alternative route by which sensory information can reach the PBN.

## Abstract 2

Supplemental Digital Content is Available in the Text.

Mouse lamina I spinoparabrachial projection neurons exhibit heterogenous electrophysiological and morphological characteristics that can be used to distinguish 4 discrete subpopulations. Lamina III-V spinoparabrachial projection neurons also exhibit defining electrophysiological properties.

## 1. Introduction

Projection neurons represent the final output of the spinal cord dorsal horn, summing sensory information from primary afferents along with more processed local circuit signals before relaying this information to the brain.^48^ Projection neurons are most abundant in lamina I but are also found in deeper dorsal horn laminae (laminae III-VI), the lateral spinal nucleus (LSN), and in lamina X.^50^ Despite their relatively widespread distribution, projection neurons account for only 5% of all neurons in lamina I, and only 1% of neurons across laminae I and II.^42^ The low incidence of projection neurons has historically made it difficult to selectively target them and study how they process and relay sensory information to their supraspinal targets in the thalamus, periaqueductal grey (PAG), parabrachial nucleus (PBN), and certain medullary nuclei.^3,10,13,31,34,36^

Electrophysiological recordings from retrogradely labelled spinal projection neurons in rat have studied action potential discharge during depolarising current step injection, describing distinct spiking patterns termed gap, and burst firing, not seen in neighbouring nonprojection neurons.^44^ Rat lamina I projection neuron spiking has also been characterised after single dorsal root stimuli, distinguishing high, medium, and low responders based on the degree of spiking.^1^ Projection neuron characterisation in the mouse is less common but recent work has identified distinct projection neuron populations encoding various intensities and modalities of sensory stimuli in labelled ascending lines for perception.^15^ Given that lamina I projection neurons display a range of electrophysiological and modality coding properties, it is highly likely that these underlie distinct roles played by functionally defined subpopulations in sensory experience. Although lamina I neurons have been the principal focus of studies into spinal projection neurons, comparatively little is known of the electrical properties and discharge characteristics of projection neurons located in the deeper dorsal horn (laminae III-V).

Our understanding of the spinal cord interneuron circuits that regulate projection neuron signalling has progressed markedly in recent year through the increased use of transgenic mice. Several interneuron-based circuits have been discovered that provide excitatory drive to projection neurons^18,27,30,39,40,46^ or mediate various forms of inhibitory control over them.^7,8,21,37^ Thus, our current understanding of dorsal horn pain processing has to incorporate data from different rodent species. This is important as previous work has reported significant differences between the rat and mouse dorsal horn. For example, the expression of the nociceptive heat transduction channel transient receptor potential vanilloid 1 (TRPV1) is much more extensive in rat,^52^ whereas the expression of the neurokinin 1 receptor (responsible for substance P responses in the dorsal horn) is more restricted in mouse.^11,41^ These differences emphasise the importance of reconciling electrophysiological data on rat projection neurons with our now extensive knowledge of local interneuron properties and circuits in mouse.

Here, we aimed to characterise the electrophysiological properties of both lamina I neurons that project to the PBN and those from deeper dorsal horn laminae, laminae III-V. We used retrograde labelling approaches to identify spinoparabrachial projection neurons (SPBNs), allowing us to compare the intrinsic properties of these cells to randomly selected unlabelled neurons (UN). We then use an unbiased cluster analysis approach to examine the properties of SPBNs in lamina I and identified distinct groups, each displaying defining combinations of electrophysiological characteristics. We also identify that SPBNs in both lamina I and in deeper laminae III-IV contribute to local network activity in the spinal dorsal horn through excitatory synaptic inputs derived from axon collaterals. Together, these data provide novel insights for pain processing mechanisms within the mouse spinal cord.

## 2. Methods

All surgical and experimental procedures were approved and undertaken in accordance with the University of Newcastle Animal Care and Ethics Committee. Experiments used wild-type C57BL/6 mice (4-8 weeks old), housed in an animal care facility with continuous access to food and water under a 12-hour light/dark cycle. To assess the distribution of mouse SPBNs, animals received unilateral injection of adeno-associated virus (AAV9-CB7∼Cl-mCherry) to the parabrachial nuclei (n = 2). This enabled the assessment of retrogradely labelled SPBNs in the contralateral and ipsilateral dorsal horn (relative to injection side). To assess neurokinin 1 receptor expression, animals (n = 4) received unilateral injection to the PBN (AAV9-CB7∼Cl-eGFP). For electrophysiological experiments, animals received bilateral injections (AAV9-CB7∼Cl-mCherry, n = 18, both sexes) in the parabrachial nuclei to maximise the spinal projection neuron number (SPBNs) for subsequent electrophysiological targeting and analysis in spinal cord slices.

### 2.1. Labelling spinoparabrachial projection neurons–intracranial viral injections

**Figure 1.**
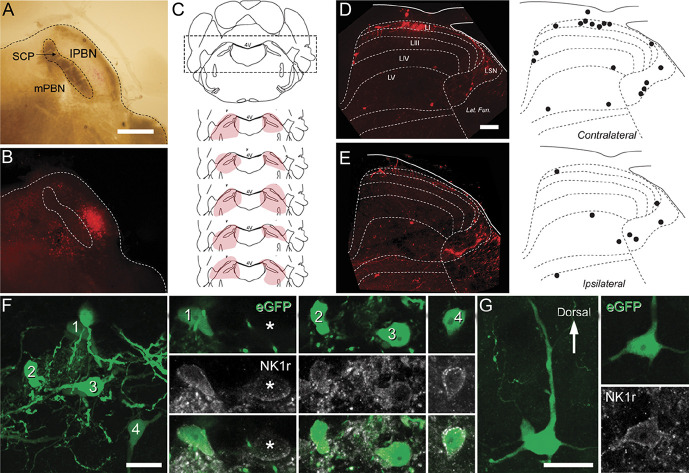
Viral labelling of mouse spinoparabrachial projection neurons (SPBNs). (A) brightfield and (B) fluorescence images show a typical injection site. The superior cerebellar peduncle (SCP) separates the medial and lateral PBN (mPBN and lPBN). In this example (B), mCherry labelling is more prominent in the lPBN than mPBN. (C) Schematic showing bilateral injection sites of AAV9-CB7∼Cl-mCherry into the PBN for retrograde SPBN labelling. Five injection sites (pink shading) are plotted on a representative coronal brain sections that includes the PBN. (D) Transverse section of the L4 spinal cord showing the distribution of retrogradely labelled mCherry SPBNs in both the contralateral (D) and ipsilateral (E) dorsal horn. Labelled cells were more abundant in the contralateral dorsal horn. On both sides, labelled cells were found in lamina I primarily, but also within the LSN and distributed more diffusely within laminae III to V. Distribution plots show the typical pattern of retrogradely labelled cells (black dots). (F and G) Closer inspection of labelled cells after injection of AAV9-CB7∼Cl-eGFP into the SPBN in lamina I (F) and lamina IV (G) showed that most SPBNs (green) showed immunolabelling for NK1 receptor (gray), but these did not account for all NK1 receptor-expressing cells in these laminae (asterisk). Scale bars (in µm): A, B = 500; D, E = 100; F, G = 20. LSN, lateral spinal nucleus.

Mice underwent surgery for injection of AAV9-CB7∼Cl-mCherry or -GFP virus into the PBN (Fig. 1). Retrograde transport, genomic incorporation, and subsequent expression of the fluorescent protein in projection neurons allowed targeted patch clamp recordings.^46^ Briefly, mice were anaesthetised with isoflurane (5% induction, 1.5%-2% maintenance) and secured in a stereotaxic frame (Harvard Apparatus, MA). Craniotomies provided access for unilateral (anatomical experiments) or bilateral (electrophysiology) PBN injection of ∼700 nL of virus through a picospritzer (PV820, WPI, FL). Injections were made over 5 minutes at stereotaxic coordinates of 5.25 mm posterior to bregma, 1.2 mm lateral to the midline, and at a depth of 3.8 mm from the skull surface according to The Mouse Brain Atlas (Paxinos and Franklin 2001). The pipette was left in place for 7 to 10 minutes after the injection to minimise drawing the virus sample along the pipette track. We adopted a 2 to 4 week postinjection recovery time to allow optimal retrograde labelling of projection neurons before spinal cord slices were prepared.^28^ All animals made an uneventful recovery and showed no overt disturbances to behaviour.

### 2.2. Tissue preparation and immunohistochemistry

For all histological studies, animals that had undergone unilateral injections to PBN were perfused transcardially with 4% depolymerised formaldehyde in 0.1M phosphate buffer 14 days after surgery.

For immunohistochemistry, transverse or sagittal spinal cord sections from unilateral PBN-injected mice (AAV9-CB7-Cl-eGFP) were processed to reveal immunolabelling for GFP, the neurokinin 1 receptor (NK1r), excitatory synapses using Homer1, and the vesicular glutamate transporter VGLUT2 as previously described (Boyle et al., 2019). Sections were incubated in cocktails of primary antibodies containing chicken anti-GFP (diluted1:1000; Abcam, United Kingdom; RRID: AB_300798), rabbit anti-NK1r (diluted 1:2000: Sigma-Aldrich, United Kingdom; RRID:AB_261562), rabbit anti-VGLUT2 (diluted 1:5000; Synaptic Systems, Germany; RRID:AB_2864778), or goat anti-Homer1 1:1k (diluted 1:1000; Frontier Institute Co. Ltd, Japan; RRID: AB_2571573), then species-specific secondary antibodies conjugated to Alexa 488 or Alexa 647. To reveal goat anti-Homer1 labelling, sections were first incubated in a biotinylated anti-goat secondary antibody raised in donkey, followed by incubation in streptavidin labelled with Pacific Blue (ThermoFisher Scientific, Waltham, MA). All antibodies were made up in 0.3 M phosphate-buffered saline with 0.3% Triton X-100.

Sections were scanned on a Zeiss LSM710 confocal microscope with Argon multiline, 405 nm diode, 561 nm solid state, and 633 nm HeNe lasers scanned through EC Plan-NEOFLUAR 10×/0.33 or Plan-APOCHROMAT ×20/0.8 lenses with zoom between 1 and 2, and z-steps of 1 µm. Confocal image stacks were analysed offline using Neurolucida for Confocal software (MBF Bioscience, Williston, VT). Cells expressing GFP were first identified, and the presence of NK1r-immunolabelling in these cells was then determined by viewing the rhodamine labelling. Because NK1r immunolabelling can be weak in some cells, expression patterns in all cells were assessed at multiple focal planes by scrolling through image stacks. Cells in the LSN were not analysed. For image presentation, the tonal range of individual channels was adjusted in projected stacks using Adobe Photoshop 10 (Adobe Systems, San Jose, CA). No adjustments were made to gamma levels.

### 2.3. Spinoparabrachial projection neuron location and distribution

The lumbosacral enlargement of AAV9-CB7∼Cl-mCherry or -GFP-injected mice was isolated and equilibrated in sucrose PBS (30% wt/vol), embedded in cryogel, and sectioned to 60 μm on a cryostat (Leica VM1900). All sections from a serial well were mounted in buffered glycerol. Sections were imaged on a Leica TCS SP8 scanning confocal microscope (25×, field of view: 445 μm × 445 μm, z-step: 1 μm). Spinoparabrachial projection neurons were counted using ImageJ plugin Cell Counter.^9^ Cells were included in the analysis if they could be observed in at least 4 serial images and were neuronal in shape. Laminae boundaries, determined using templates derived from the Allen Mouse Brain Atlas (http://mousespinal.brain-map.org/imageseries/showref.html), were superimposed over the dorsal horn images and SPBNs assigned by their location as laminae I-V and LSN. Brainstems from these animals were embedded in 4% agarose in 0.1M phosphate-buffered saline and sectioned at 100 μm to confirm the injection site. Only tissues from animals with clear evidence of injection sites localized to PBN were included for analysis.

### 2.4. Spinal cord slice preparation

Spinal cord slices for patch clamp electrophysiology were prepared using previously reported methods.^46^ Briefly, animals were anaesthetized with ketamine (100 mg/kg *i.p*.) and decapitated. The spinal cord was rapidly isolated in ice-cold sucrose substituted cerebrospinal fluid (ACSF) containing (in mM): 250 sucrose, 25 NaHCO_3_, 10 glucose, 2.5 KCl, 1 NaH_2_PO_4_, 1 MgCl_2_, and 2.5 CaCl_2_. Tissue was prepared in either transverse (LI-L5 segments, 300 μm thick) or sagittal slices (L1-L5 segments, 200 µm thick) using a vibrating microtome (frequency: 65 Hz, amplitude: 2.00 mm, 0.05 mm/s; Campden Instruments 7000 smz, Loughborough, United Kingdom). Slices were transferred to an interface incubation chamber containing oxygenated ACSF (same composition as sACSF except 118 mM NaCl substituted for sucrose) and allowed to equilibrate at room temperature for at least 1 hour before recording. In an initial set of recordings, the brainstems from injected animals were removed, fixed overnight in (4% PFA in 0.1M PB), and sectioned to assess mCherry expression at the injection site (Figs. 1A–C). In subsequent experiments, unfixed brains were sectioned immediately after spinal cord slicing and immediately checked to verify red fluorescent protein signal within PBN. Analysis is only included for animals with clear mCherry expression within the PBN of the brainstem sections.

### 2.5. Patch clamp electrophysiology

Spinal cord slices were recorded in a chamber and continuously superfused with ACSF, bubbled with carbanox (95% O_2_, 5% CO_2_) to achieve a final pH of 7.3 to 7.4. All recordings were made at room temperature (22-24 °C). Retrogradely labelled SPBNs in lamina I, or less frequently laminae III-V, were identified by mCherry expression and targeted for whole-cell patch clamp recording. In addition to SPBNs, a sample of unlabelled neurons (UN) in the same region as mCherry-labelled SPBNs was recorded for comparison. As the PBN injections have previously been reported to capture most projection neurons (5% of LI),^11^ these LI UN recordings were most likely local LI interneurons. Patch pipettes (4-8 MΩ; Harvard glass) were filled with a potassium gluconate-based internal solution containing (in mM): 135 C_6_H_11_KO_7_, 8 NaCl, 10 HEPES, 0.1 EGTA, 2 Mg_2_ATP, and 0.3 Na_3_GTP, pH 7.3 (with KOH). No liquid junction potential correction was made, although this value was calculated at 14.7 mV (22 °C). Neurobiotin (0.2% wt/vol) was included in the internal solution for SPBN recordings, to allow post hoc analysis of cell morphology. All data were collected using a MultiClamp 700B amplifier (Molecular Devices, Sunnyvale, CA), digitized online (sampled at 10 kHz, filtered at 5 kHz) using an ITC-18 computer interface (Instrutech, Long Island, NY), and acquired and analysed using AxoGraph X software (Molecular Devices, Sunnyvale, CA).

Action potential (AP) discharge was assessed as previously reported in current clamp mode from a membrane potential of −70 mV,^9^ maintained by injecting small bias currents when necessary (±20 pA). A series of depolarising current steps evoked AP discharge (20 pA increments, 1 second duration), which was subsequently classified based on previously described work in rat lamina I projection neurons,^44^ overlapping with existing schema for lamina II neurons.^22–24^ Classifications were based on the voltage response recorded 2 steps above the first current injection to evoked AP discharge (ie, rheobase + 40 pA). Gap firing (GF) featured an initial spike at onset, followed by a ramped voltage response before additional AP discharge resumed; delayed firing (DF) exhibited a clear ramped voltage response before AP discharge; tonic firing (TF) was characterised by sustained AP discharge; initial bursting (IB) exhibited a burst of AP discharge at current onset often with an underlying depolarising hump at rheobase; single spiking (SS) featured a single AP at onset, regardless of step amplitude; phasic (P) discharge showed periods of discharge interrupted by breaks throughout current step injection. Reluctant firing (RF) was assigned to neurons that lacked AP discharge despite sustained depolarisation up to ∼20 mV above the AP threshold. Importantly, responses were only deemed RF if neurons did fire APs when the step protocol was repeated from a more depolarised membrane potential.^22^

Subthreshold currents underlying AP discharge were assessed using a voltage-clamp protocol with an initial hyperpolarisation step −70 to −100 mV (1 second duration), followed by a depolarizing step to −40 mV (200 ms duration), with P/N leak subtraction applied. Four major voltage-activated currents were identified, including fast and slow forms of A-type outward potassium currents (I_A_), low-threshold transient inward currents with T-type calcium current characteristics^12^ (Ca_T_-like), and a nonspecific inward cationic current commonly referred to as I_h_.^23^ Spontaneous excitatory postsynaptic currents (sEPSCs) were recorded (holding potential −70 mV). Input and series resistance (<40 MΩ) were monitored throughout all recordings, and data were excluded if these values changed by more than 10%.

### 2.6. Patch clamp data analysis

Data were analysed offline using AxoGraph X software as previously described. Membrane capacitance and input resistance were calculated (averaged response to -5mV step, 30 trials, holding potential −70 mV). Resting membrane potential was taken as the average of 30 seconds of passive current clamp recording (bias current = 0 pA). A cell was classified as spontaneously active if any APs were observed more than 60 seconds of passive current clamp recording and classified by previously described criteria.^33^ AP threshold (point when dV/dt was 15 mV/ms) was taken from the rheobase response, and the first AP generated at the rheobase + 40 pA step response was used to determine the following: AP peak (difference between the maximum positive peak and AP threshold); AP rise time (duration between AP threshold and peak AP); AP base width (measured at AP threshold); afterhyperpolarisation (AHP) amplitude (difference between AP threshold and maximum negative deflection); and AHP latency (time between AP threshold and the maximum negative peak). The AHP profile was also classified into 3 categories based on previous work in rat.^44^ These included AHPs with: monophasic return to baseline with a simple time course; pronounced afterdepolarisation before returning to baseline; and distinctly slowed falling AP phases in repolarisation occasionally appearing as a prominent hump. In those cells that exhibited repetitive AP discharge, spike latency was the time from current step onset to the first AP threshold, interspike interval was the time between successive AP peaks, instantaneous frequency was the reciprocal of interspike interval, mean frequency was calculated from the number of APs elicited during a depolarising current step, discharge duration was the time between the first and last APs in a response, AP adaptation was the ratio of the first and last APs' instantaneous frequency, and AP attenuation was the ratio of the first and last AP peak.

Spontaneous excitatory postsynaptic currents were analysed using a sliding template detection method, and average sEPSC frequency determined over at least 30 seconds. Peak sEPSC amplitude, rise time (10%-90% of peak), and decay time constant (Tau; 10%-90% of the decay phase) were obtained from averaged sEPSCs. Spontaneous excitatory postsynaptic current charge was the area under the curve of an averaged sEPSC, and excitatory synaptic drive was calculated by multiplying sEPSC charge with sEPSC frequency. For subthreshold responses, the fast (I_Af_) and slow (I_As_) I_A_ currents were distinguished by the latency to peak outward current, (fast, <15 ms; slow, >15 ms). I_h_ was identified as a ramped sag current step during hyperpolarisation. Low-threshold, fast activating inward currents consistent with T-type calcium currents (I_Ca_-like) were identified as transient inward currents. For both I_A_ currents and I_Ca_-like currents, peak amplitude was measured as the maximum current evoked during the −40 mV step. I_A_ and I_Ca_-like current latency was the time from −40 mV step onset to peak current, and 50% decay was the time between the maximum peak and half-current amplitude in the decay phase.

### 2.7. Morphological characterisation of spinoparabrachial projection neurons

Slices containing recorded neurons filled with Neurobiotin were incubated in streptavidin-Cy5 (1:50: Jackson ImmunoResearch Laboratories Inc; RRID AB_2337245) for 2 hours. Overlapping tiled confocal scans captured the entire somatodendritic arborisation of labelled cells at 25× magnification on a Leica TCS SP8 scanning confocal microscope (z-step = 1 μm; field of view 445 μm × 445 μm; pinhole = 1 AU). Confocal image stacks were analysed offline using the open source image processing software FIJI.^45^ Each recovered SPBN and its dendritic territory was assessed in rostrocaudal, dorsoventral, and mediolateral planes. The soma location of each recovered SPBN was differentiated as medial, central, or lateral in transverse slices by dividing the dorsal horn into 3 equal regions. Soma location was differentiated in sagittal slices as medial, middle, and lateral using overall slice appearance. Medial slices were distinguished by bundles of myelinated afferents passing into the dorsal gray matter. Slices from the lateral region were identified by a prominent fibre tract parallel to the dorsal slice surface and located ventral to the dorsal gray matter. Slices were classified as middle dorsal horn when medial and lateral slice features were absent. Dendritic territory was measured from the centre of a filled neuron's somata using 3D image stacks. For neurons recovered in sagittal slices, rostrocaudal length was defined as the distance between the rostral and caudal extremities of labelled dendrites originating from the cell body, measured parallel to the dorsal edge of the slice. Dorsal and ventral lengths were defined as the distance between dendrites in continuity with the cell body in each respective direction, measured perpendicular to the white/gray matter border. Dorsoventral length was the sum of these dorsal and ventral measurements. Mediolateral values were not assessed as neuronal processes in this plane and would have been limited by slice thickness (300 μm). The same approach was taken for transverse slices, except medial and lateral lengths were determined as the distance of the longest terminal dendrites in each direction, measured parallel to the dorsal slice edge. These values were also summed to provide a mediolateral length. Dorsal, ventral, and dorsoventral lengths were measured identically to sagittal slices. Rostrocaudal length was not determined in transverse slices as it would have been limited by slice thickness (300 μm). In some neurons, axons could be identified by their thin, constant diameter (no taper) profile, varicosities, and variable paths.^47^ Branching points along recovered axons were assessed to provide evidence of local collateral branches within the dorsal horn, as previously described in other species.^5,6,47^

### 2.8. Clustering analysis parameters

To compare the electrophysiological properties of SPBN and UN groups, a hierarchical clustering approach was used to determine whether groups or subpopulations existed among the cells sampled. Clustering analyses were completed using Orange v3.2 data analysis software^17^ and used only electrophysiological data to maintain the largest possible sample, given morphology was only recovered in a subset of recordings. All electrophysiological parameters were imported, and Euclidean distance was calculated for these values. A hierarchical cluster analysis was then completed using Ward's linkage. Dendrograms were generated for cluster analysis outputs and heatmaps produced for each parameter. Colour range was scaled to maximum and minimum values for each property. Cluster number was determined using the inbuilt silhouette scores, which takes into account the distance between clusters. The number of clusters was assigned by selecting the point where additional clusters introduced negative silhouette scores, indicating that adding further clusters does not account for variability in the data.

### 2.9. Statistical analysis

All data are presented as mean ± SD unless otherwise stated. Unpaired *t* tests were used to compare SPBN with UN populations in lamina I and SPBNs located in lamina I with those in laminae III-V. The χ^2^ tests compared the distribution of AP discharge patterns, AHP profile, and subthreshold currents among groups. One-way analyses of variance were used to compare differences between identified SPBN clusters, and the Tukey post hoc test was used to determine which clusters differed.

## 3. Results

Targeted recordings were made from 79 lamina I (LI) SPBNs, collected from spinal cord slices cut in either the sagittal (n = 17) or transverse (n = 62) plane. A complementary data set of unidentified lamina I neurons was also collected for comparison (n = 26; 24 transverse and 2 sagittal). Finally, targeted recordings were also made from SPBNs located in laminae III-V (n = 13; 8 transverse and 5 sagittal).

### 3.1. Distribution of labelled spinoparabrachial projection neurons

The distribution of spinal SPBN labelling was assessed in tissue from the unilateral injection of AAV9-CB7-Cl-mCherry (Figs. 1D–E, n = 2). This captured the most SPBNs in LI of the contralateral dorsal horn (presented as total counted SPBN, and mean ± SD: LI: animal A: 115, 8.2 ± 3.6 neurons/60 μm, animal B: 63, 6.3 ± 2.9 neurons/60 μm), followed by the LSN (animal A: 40, 2.9 ± 1.4 neurons/60 μm; animal B: 12, 1.2 ± 0.9 neurons/60 μm), LV (animal A: 40, 2.9 ± 1.4 neurons/60 μm; animal B: 8, 0.8 ± 0.8 neurons/60 μm), and LIII/LIV (animal A: 16, 1.1 ± 0.9 neurons/60 μm; animal B: 10, 1 ± 0.7 neurons/60 μm). Spinoparabrachial projection neurons were also observed on the ipsilateral dorsal horn, but in fewer numbers (LI: animal A: 29, 2 ± 1.5 neurons/60 μm, animal B: 12, 1.3 ± 0.6 neurons/60 μm; LSN: animal A: 28, 2 ± 1 neurons/60 μm, animal B: 13, 1.3 ± 1.3 neurons/60 μm; LV: animal A: 36, 3 ± 1.9 neurons/60 μm, animal B: 10, 1.0 ± 0.7 neurons/60 μm; and LIII/LIV: animal A: 3, 0.2 ± 0.6 neurons/60 μm, animal B: 4, 0.4 ± 0.7 neurons/60 μm) (Figs. 1D, E).

### 3.2. Neurokinin 1 receptor expression in most viral-labelled mouse spinoparabrachial projection neurons

A total of 182 GFP-labelled cells in laminae I-IV were analysed from 4 animals (16, 78, 6, and 83 cells per animal). All cells analysed were contralateral to the injection site, although cells were also seen in the ipsilateral dorsal horn. Of the 170 SPBNs identified in lamina I (12, 75, 5, and 78 per animal), 141 showed immunolabelling for NK1r (n = 141/170, 83%, 10/12, 67/75, 2/5, and 62/78) (Fig. 1F). A total of 12 SPBNs were identified in deeper dorsal horn laminae, with 5 of these showing immunolabelling for NK1r (n = 5/12, 42%, 1/4, 2/3, 1/1, and 1/5 per animal) (Fig. 1G).

### 3.3. Electrophysiological properties of lamina I spinoparabrachial projection neurons and unidentified neurons

There was little difference between LI SPBNs and UNs in passive membrane properties, such as input resistance (295.4 ± 191 vs 289 ± 162.4 MΩ, *P* = 0.89), resting membrane potential (−56.5 ± 7.2 vs −56.4 ± 9.6 mV, *P* = 0.89), or rheobase current (50.6 ± 38.3 vs 45.4 ± 28.6 pA, *P* = 0.52), respectively. We did, however, observe that the mean membrane capacitance of LI SPBNs was greater than that of UNs (13.5 ± 4.4 vs 10.8 ± 3.7 pF, *P* = 0.01). This is consistent with projection neurons being larger than UNs.^2^ Spontaneous AP discharge, defined as the presence of AP spiking during 1 minute of recording, was also significantly higher in LI SPBNs than that in UNs (47% vs 20%, *P* = 0.02). Although only approximately half the SPBN sample exhibited spontaneous activity, those that were active showed mostly irregular spiking, with remaining cells showing tonic activity (80% and 20%, respectively). No recordings exhibited bursting spontaneous activity as previously described for neonatal SPBNs.^33^

Given that the rheobase current was similar in both LI SPBNs and UNs, we next compared SPBN and UN AP properties. These comparisons showed that the AP threshold was lower in LI SPBNs (−39.2 ± 5.8 vs −31.6 ± 8.3 mV, *P* < 0.001), AP peak amplitude was larger (55.3 ± 13.5 vs 39.6 ± 13.5 mV, *P* < 0.001), and rise time was shorter (2.17 ± 0.93 ms vs 2.9 ± 1.0 ms, *P* = 0.001) than those in UNs. By contrast, AP width (5.6 ± 2.0 vs 6.1 ± 2.1 ms, *P* = 0.31) and AHP peak amplitude (−19.2 ± 5.0 vs −19.8 ± 5.6 mV, *P* = 0.64) were similar in both groups; however, AHP peak amplitude occurred at a longer latency in LI SPBNs (15.6 ± 11.9 vs 11.6 ± 4.4 ms, *P* = 0.014). Together, these data suggest that LI SPBNs cannot be confidently distinguished from UNs based on their passive membrane properties. By contrast, some active properties did differ between cell types, with LI SPBNs expressing properties consistent with a more easily recruited population than the UN sample.

### 3.4. Discharge properties

**Figure 2.**
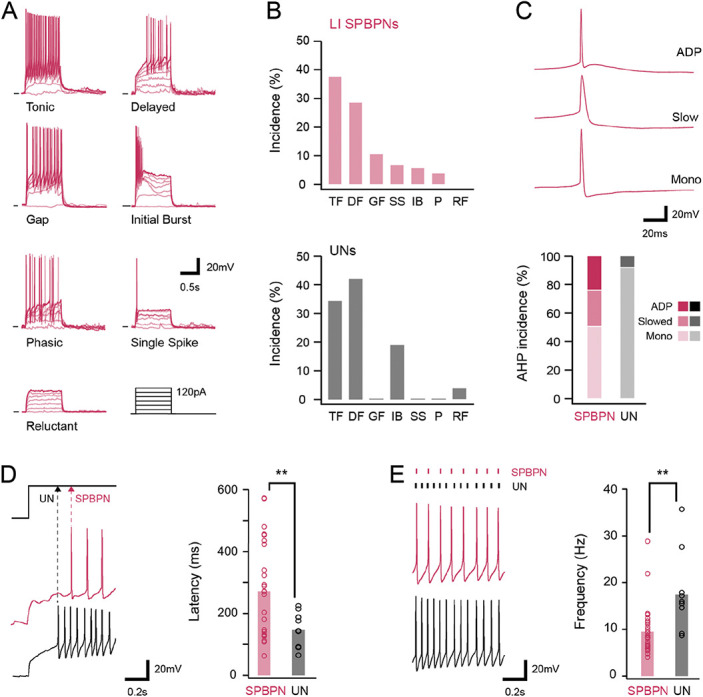
Action potential discharge characteristics of LI SPBNs and unidentified neurons (UNs). (A) Traces show overlaid recordings of SPBN membrane potential during depolarising current step injections (lower right black traces). Responses were classified as follows: tonic firing (TF), delayed firing (DF), gap firing (GF), initial bursting (IB), phasic (P), single spiking (SS), and reluctant firing (RF). (B) Plots show the incidence of different discharge patterns in SPBNs and UNs. Note, both populations exhibited similar levels of TF; however, GF, P, and SS were only observed in SPBNs, and DF was more common in UNs. (C) Traces show APs on an expanded time scale to highlighting 3 SPBN afterhyperpolarisation profiles. Some recordings included an afterdepolarisation phase during AP repolarisation (upper), other recordings exhibited a slowed repolarisation phase that extended AP width (middle), and many recordings showed a monophasic afterhyperpolarisation phase (lower). Bar plot (below) summarise the incidence of afterhyperpolarisation phases in LI SPBNs and UNs. (D) Traces show the membrane potential recorded from a LI SPBN and UN during the onset of depolarizing step injection (step shown above). Dashed lines highlight different delays between LI SPBNs and UNs. Group data plot (right) compares the latency to first action potential spike in LI SPBN and UN samples. (E) Traces compare the AP discharge mid depolarizing step in LI SPBNs and UNs, dashes above highlight different spike frequencies between traces. Group data plot (right) compares the mean action potential discharge frequency (right) showing SPBNs discharge APs at lower frequencies than UNs. AP, action potential; SPBNs, spinoparabrachial projection neurons.

We next assessed responses to depolarising current step injections (Fig. 2A), initiated from a membrane potential of −70 mV (sample average: −68.9 mV). The response to these current steps of increasing amplitude was used to characterise each neuron's “discharge pattern” response, as previously reported for other DH populations in vitro and in vivo.^22–24,44,51^ For LI SPBNs, 6 types of discharge were differentiated (Fig. 2B) with TF the most prevalent (∼37%), followed by DF (∼29%), GF (∼13%), and IB (∼7%) responses. A small proportion of LI SPBN neurons exhibited SS and P responses (∼8%, and ∼5%, respectively) (Fig. 2A). By contrast, LI UNs exhibited 4 types of discharge (Fig. 2B lower panel) with DF the most prevalent (42%), followed by TF (∼35%), IB (∼19%), and RF (∼4%). These UN distributions are consistent with many reports in the literature.^9,22–24,44,51^

The profile of repolarization and AHP phase also differed between samples (Fig. 2C), with LI SPBNs exhibiting varied AHP profiles including monophasic repolarisation, slowed repolarizations, and afterdepolarisations (n = 40/79, n = 20/79, and n = 19/79, respectively). Furthermore, most afterdepolarisations were superimposed on a slow hyperpolarisation phase (n =13/19, 68.4%). By contrast, UNs exhibited mostly monophasic repolarisations, limited examples of slowed repolarization, and no afterdepolarisations (n = 23/25, n = 2/25, n = 0/25; SPBN vs UN: 50.6% vs 92.0%, 25.3% vs 8.0%, and 24.1% vs 0%, respectively: *P* = 0.001). When responses to current steps were compared across the LI SPBN and UN samples, regardless of discharge patterns, it was clear that LI SPBNs generally discharged APs at lower rates and better maintained peak AP amplitude during repetitive firing. This was reflected in a lower average instantaneous firing frequency for SPBNs (11.7 ± 8.6 vs 16.8 ± 9.1 Hz, *P* = 0.01), fewer APs elicited during current steps (8 ± 5 vs 11 ± Hz, *P* = 0.04), and reduced AP peak attenuation (last AP peak/first AP peak: 76.6 ± 17.6 vs 55.6 ± 21.3%, *P* < 0.001). By contrast, other discharge features such as the first AP latency (125.6 ± 148.1 vs 88.5 ± 71.6 ms, *P* = 0.10), AP discharge duration (665 ± 293 vs 660 ± 323 ms, *P* = 0.93), and AP frequency adaptation (0.77 ± 0.34 vs 0.68 ± 0.27, *P* = 0.14) were statistically similar for both cell types.

As the above findings may be influenced by differences in the incidence of discharge patterns in the LI SPBN vs UN samples, AP discharge was also compared within the discharge categories common to both sample (Figs. 2D, E). This comparison resolved differences in DF with the latency to first spike longer in the LI SPBN population (271.5 ± 161 vs 146.6 ± 53 ms, *P* = 0.001) than that in UNs (Fig. 2D). Consistent with the population-wide comparison (above), TF responses in LI SPBNs also exhibited lower instantaneous frequencies (Fig. 2E) and included fewer APs during each current step (9.6 ± 5.1 vs 18.0 ± 8.6 Hz, *P* = 0.001; 9.4 ± 5.0 vs 17.2 ± 8.7 APs, *P* = 0.002). Finally, the presence of an initial depolarising hump (preceding AP discharge) was a feature of LI SPBNs but rare in UNs (48% vs 8%, *P* = 0.018). Together, this analysis suggests that differing ionic currents influence discharge phenotypes in the SPBN and UN populations.

### 3.5. Dominant subthreshold currents in lamina I spinoparabrachial projection neurons

**Figure 3.**
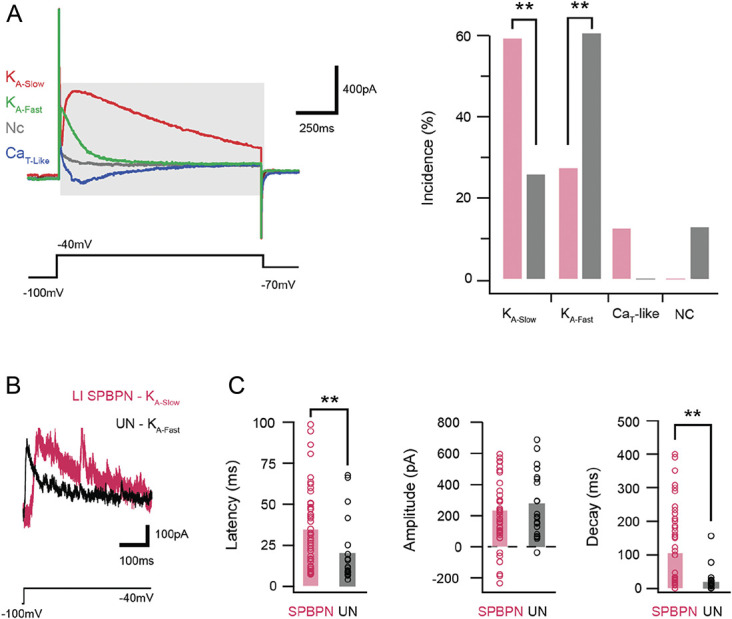
Subthreshold current expression in LI SPBNs and unidentified neurons. (A) Overlaid traces show subthreshold voltage-activated currents in responses to a voltage step protocol (lower black trace). Four characteristic responses are shown and include a fast A-type potassium current (K_A-Fast_, green), a slow A-type potassium current (K_A-Slow_, red), a T-type-like low-threshold calcium current (Ca_T_-like, blue), or no current in a passive response (NC, gray). Plot (right) compares the incidence of subthreshold currents in SPBNs and UNs. Note, LI SPBNs exhibit greater K_A-slow_ and Ca_T_, whereas K_A-Fast_ dominates in UNs and these cells do not exhibit Ca_T_ currents. (B) Traces show A-type potassium currents recorded from a LI SPBN and UN on an expanded time scale following P-N subtraction. Note, the different time courses of K_A-Slow_ and K_A-Fast_. (C) Group data plots compare latency, amplitude, and decay time course of currents activated by a voltage step to −40 mV in LI SPBNs and UNs. Consistent with a greater incidence of K_A-Slow_ in LI SPBNs, these neurons show longer latency and slower decay kinetics. In addition, a number of LI SPBNs show inward (negative) peak currents indicating Ca_T_-like currents. SPBNs, spinoparabrachial projection neurons; UN, unlabelled neurons.

Our analysis of subthreshold voltage-activated currents showed that most LI SPBN and all UN neurons exhibited outward K currents (Fig. 3A, 86% vs 100%, respectively, 65/76 and 26/26). By contrast, only lamina I SPBNs exhibited the Ca_T_-like inward currents (n = 9/76). Of the cells exhibiting A-type potassium currents, these could be further subdivided into slow or fast variants (K_A-Slow_ and K_A-Fast_). Lamina I SPBNs were more likely to exhibit the K_A-Slow_ (61% vs 23%, 46/76 and 6/26, *P* = 0.001, Fig. 3B), whereas the opposite was true for UNs, with K_A-Fast_ dominating (25% vs 62%, n = 19/76 and 16/26, *P* = 0.001, Fig. 3B). When quantifying the mean amplitude of current responses evoked by the −40 mV step (SPBN: n = 47, UN: n= 20, respectively), regardless of polarity or time course, there was no difference between LI SPBNs and UNs. By contrast, the rise time (Fig. 3C) (35.85 ± 24.4 vs 20.3 ± 20.02 ms, *P* = 0.015) and decay kinetics (half-width: 131.4 ± 119 vs 47.2 ± 69 ms, *P* < 0.001) of these currents were slower in LI SPBNs (Fig. 3C). This is consistent with the dominance of the K_A-Slow_ current in SPBNs. In addition to depolarisation-activated currents, the hyperpolarising voltage step from −60 mV to −120 mV identified a slowly activating hyperpolarisation-activated cation current, (I_h_) in some recordings. When present, both the incidence (LI SPBN: 25% vs UN: 54%) and the peak amplitude (16.1 ± 11.7 pA vs 22.3 ± 6.8 pA, *P* = 0.01) of I_h_ currents differed between the 2 populations, being less prevalent and smaller in LI SPBNs. Together, these results show that subthreshold currents in LI SPBN generally act to restrict cell excitability (K_A-slow_), by counteracting depolarisation and in some cases SPBNs exhibit currents that counteract hyperpolarisation (ie, I_h_).

### 3.6. Spontaneous excitatory synaptic drive in LI spinoparabrachial projection neurons

**Figure 4.**
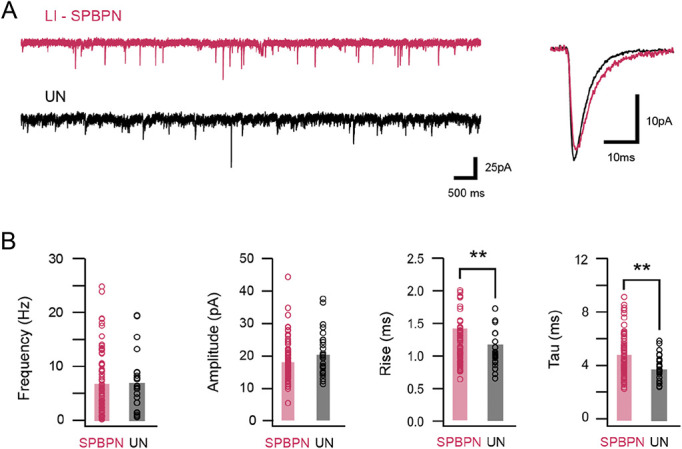
Spontaneous excitatory synaptic input characteristics in SPBNs and unidentified lamina I neurons. (A) Traces (left) show continuous spontaneous excitatory synaptic current (sEPSC) recordings from a LI SPBN (red) and UN (black). Overlaid traces (right) compare averaged sEPSCs taken from the same cells. Note, currents have similar amplitudes, but LI SPBNs exhibit a slower time course. (B) Group plots compare average sEPSC frequency, amplitude, rise time, and decay time constant in LI SPBNs and UNs. Consistent with examples in (A) the frequency and amplitude of sEPSCs are similar between the 2 neuron types, but the rise time and decay time constant are slower in SPBNs. SPBNs, spinoparabrachial projection neurons; UN, unlabelled neurons.

**Figure 5.**
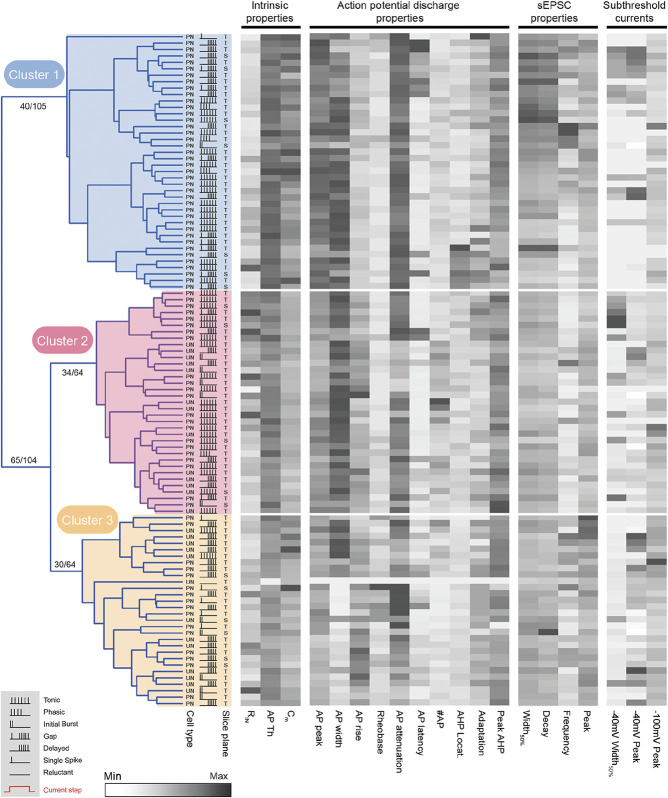
Hierarchical clustering of SPBNs and UNs. Dendrogram and heatmaps plot the assignment of SPBN and UN into clusters based on their electrophysiological properties. The AP discharge pattern of each neuron is annotated using the key in the lower left gray box (inset). The slice orientation of each recording is indicated as transverse or sagittal (T or S, respectively), and identity is denoted as SPBN or UN (PN or UN, respectively). Dendrograms show clusters (3 in this analysis) determined using K-Means-based silhouette scoring. Heatmaps present the relationship of 20 electrophysiological characteristics, normalised to maximum (black) and minimum (white) values for each property, aligned to each neurons cluster assignment. This analysis reveals one cluster of pure SPBNs (cluster 1, blue, n = 40), and 2 mixed clusters where SPBN and UN subpopulations exhibited similar properties (cluster 2, red, n = 35; and cluster 3, yellow, n = 30). AP, action potential; PN, projection neuron; SPBNs, spinoparabrachial projection neurons; UN, unlabelled neurons.

Ongoing spontaneous excitatory synaptic drive was assessed and compared in LI SPBNs and UNs (Fig. 4A). Spontaneous excitatory postsynaptic current frequency (6.75 ± 5.8 ± vs 6.75 ± 5.1 Hz, *P* = 1.0), peak amplitude (−21.0 ± 6.3 vs −23.0 ± 7.0 pA, *P* = 0.16), and charge (1077.0 ± 1169 vs 883.4 ± 793.5 pA.s, *P* = 0.70) were similar in LI SPBNs and UNs (Fig. 4B, left plots). However, sEPSC kinetics differed in the 2 populations (Fig. 5A, right traces) with slowed decay kinetics (Tau = 5.9 ± 2.1 vs 4.5 ± 1.1 ms, *P* < 0.001), and rise times (1.43 ± 0.41 vs 1.18 ± 0.30, *P* = 0.005) in LI SPBNs compared with UNs. Together, these data indicate that overall excitatory drive is similar in the 2 populations; however, their differing kinetics suggest the involvement of different ligand-gated channel subtypes and/or differing distribution of excitatory inputs across each populations somatodendritic trees.

As slice orientation varied between transverse and sagittal orientations, influencing the retention of dendritic arbors and potentially sEPSCs, we also separated and compared recordings in each slice orientation. This comparison did not detect differences in sEPSC decay time (6.29 ± 2.8 ms vs 5.76 ± 1.74 ms, *P* = 0.48), frequency (7.95 ± 6.2 Hz vs 6.45 ± 5.7 Hz, *P* = 0.362), and amplitude (17.13 ± 4.8 pA vs 18.73 ± 6.4 pA, *P* = 0.35) recorded from SPBNs in the sagittal plane (n = 17) vs transverse recordings (n = 62). This suggests that when recording from the soma, the contribution of sEPSCs in the distal dendrites, potentially removed in transverse slices, is low compared with synapses closer to the soma that would be better preserved in both orientations.

### 3.7. Unsupervised clustering of spinoparabrachial projection neurons and UNs segregate distinguishable clusters

To test for the overall selectivity of SPBN properties compared with UNs, key active and passive properties were used to perform an unsupervised hierarchical cluster (Fig. 5); yielding 3 distinct clusters. Strikingly, one cluster was purely SPBNs (Cluster 1: 40/40, 100% purity), and the 2 remaining clusters contained a mixture of SPBNs and UNs (Cluster 2: 22/35, ∼65%; Cluster 3: 17/30, ∼57%). Not surprisingly, these clusters were separated by factors that differed in SPBN and UN populations, including intrinsic membrane, AP, and sEPSC characteristics (Supplementary Table 1, available at http://links.lww.com/PAIN/B260). For example, Cluster 1 exhibited AP discharge patterns with rapid and longer responses (ie, TF: 42.5%, GF: 20%, IB: 2.5, PF: 3%, n = 29/40, 72.5%), less delayed responses (DF: 25%, SS: 2.5%, n = 11/40, 27.5%), and had the highest proportion of GF. Cluster 2 showed similar distributions to Cluster 1 (TF: 60%, IB: 14.3%, GF: 5.7%, PF: 2.9%, n = 29/35, 82.9%), with the remainder showing DF (n = 6, 17.1%). By contrast, patterns characterised by more difficult to recruit AP discharge were more strongly represented in Cluster 3 (DF: 56.7%, SS: 16.7%, RF: 3.3%, 23/30, 76.7%) with the remainder mixed between IB and tonic discharge responses (IB: 16.7%, TF: 6.7%). Together, this analysis reinforces the existence of a distinct SPBN group with high membrane capacitance, large soma size, low input resistance, longer time course sEPSCs, and increased incidence of Gap AP discharge patterns. At the same time, some SPBNs differed from this group, clustering the subsets of UNs.

### 3.8. Somatodendritic characteristics of recorded lamina I spinoparabrachial projection neurons

**Figure 6.**
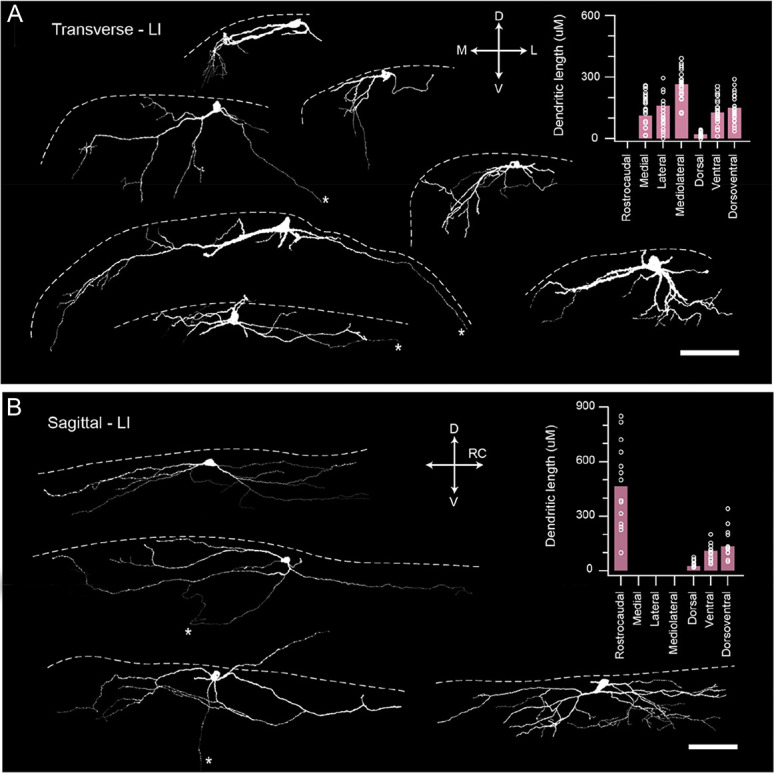
Morphological properties of SPBNs. (A) Images show examples of LI SPBN morphology recovered from transverse spinal cord slices after patch clamp recordings (dashed white line denotes position of dorsal gray/white matter border). Red image shows a Neurobiotin-recovered LI SPBN, with traced cell below (white). Several traced cells show the diversity of LI SPBN morphologies. Group data plot (right) summarizes LI SPBN dendritic dimensions in the rostrocaudal, mediolateral, and dorsoventral planes. Note: rostrocaudal measurements were not included because of tissue slice orientation. (B) Images show examples of LI SPBN morphology recovered from sagittal spinal cord slices after patch clamp recordings. Red image shows a Neurobiotin-recovered cell (maximum intensity z projection) with traced image below, as in (A). Several traced cells show the range of LI SPBN morphologies. Group data plot (right) summarizes LI SPBN dendritic dimensions as in (A). Note: mediolateral dimensions were excluded because of the orientation of the slice preparation. (scale A and B: 100 μm). SPBNs, spinoparabrachial projection neurons.

The above electrophysiological analysis was based on recordings from a total of 79 lamina I SPBNs, of which ∼50% (39/79) were suitably filled with Neurobiotin and recovered for morphological analysis. This included 25 cells from transverse slices (Fig. 6A) and 14 cells from sagittal slices (Fig. 6B). Most recovered LI SPBNs were located in the middle third of the dorsal horn (20/39: 51.2%), followed by the medial third (14/39: 35.9%), and finally the lateral third (5/39: 12.8%) of the dorsal horn. The mean soma size of these cells, taken as maximal cross-sectional area, varied greatly (mean = 221 μm^2^ ± 105, range = 99–550 μm^2^). Dendritic territories were measured in orthogonal planes (mediolateral, dorsoventral, and rostrocaudal) except for 2 cells in the lateral DH where the curved nature of the gray/white matter boundary and associated cell orientation had the potential to artificially affect this analysis, particularly in the dorsoventral axis. For LI SPBNs in transverse slices (Fig. 6A) the mean mediolateral dendritic length was 262 μm (±67), with the dendritic tree being biased to the medial aspect of the dorsal horn (156 μm ± 74 vs 107 μm ± 71, medial vs lateral), yielding an M/L ratio of 7.3. The mean dorsoventral dendritic length was also biased, extending more prominently into ventral/deeper laminae (DV total = 149 ± 66 μm; 21 ± 20 μm vs 128 ± 68 μm, dorsal vs ventral), with an average D/V ratio of 0.31. In line with this observation, there were several examples of dendrites extending ventrally beyond LI in our sample (>30 um ventral), although we did not assess the laminar location of these dendrites, which would have required additional neurochemical labelling for boundary demarcations. As noted above, rostrocaudal dendritic length was not assessed in transverse slices. This was addressed by also examining LI SPBNs recovered in sagittal slices (Fig. 6B), where dendritic arborisations in the rostrocaudal plane were extensive (n = 14; 444.3 ± 230 μm). As observed in transverse slices, there was also a bias to ventrally projecting LI SPBN dendrites in sagittal slices (DV total = 135 ± 65 μm; 25 ± 22 μm vs 110 ± 63 μm, dorsal vs ventral), with a D/V ratio of 0.38. There was one example of a SPBN with LI located soma that showed a clear dendrite ascending into the overlying white matter over 100 μm. The mediolateral dendritic length was not assessed in sagittal slices. Together, these measurements show that mouse LI SPBNs have extensive dendritic trees oriented in all planes. This would enable them to receive inputs from multiple laminae and across spinal cord segments.

### 3.9. Mouse spinoparabrachial projection neurons give rise to local axon collaterals within the superficial dorsal horn

**Figure 7.**
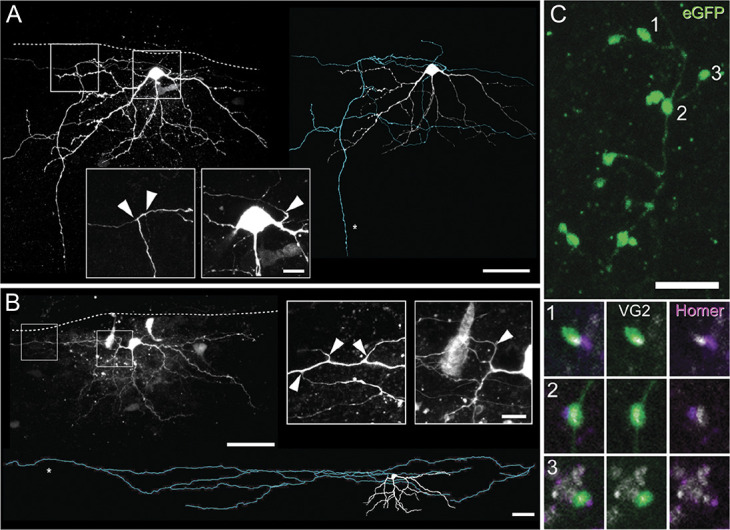
Local axon collaterals arising from mouse SPBNs. (A and B) Images show examples of LI SPBN axon recovered from sagittal spinal cord slices after patch clamp recordings (dashed white line denotes white/gray matter border). A thin, consistent diameter process emanating from either the soma or a stem dendrite was classified as an axon. These axons were seen to give rise to collateral branches. A and B show maximum intensity z projections of a Neurobiotin-filled SPBNs (left) showing clear somatodendritic and axonal labelling (denoted by *). Scale bars = 50 μm and 10 μm for insets. Traced reconstruction of the SPBNs in A and B are also shown with somatodendritic profiles in white and axonal profiles in green to fully summarise the neuronal morphology and axonal territory. Insets show clear axonal branching points within the dorsal horn (left) and axons originating from the soma or a primary dendrite (right, arrows). (C) GFP-labelled SPBN axon including *en passant* boutons (arrows). Lower panels show high-magnification images of GFP expressing axon collaterals (from above) with vesicular glutamate transporter 2 (white) and Homer immunolabelling (purple). Vesicular glutamate transporter 2 is co-expressed in axon collaterals (middle) and closely apposed by Homer puncta (right) confirming functional synapses. SPBNs, spinoparabrachial projection neurons.

Of the 39 SPBNs that were sufficiently recovered for morphological analysis, we found 20 cells with an intact axon. In 11 of the 20 recoveries, the axon originated directly from the soma, whereas the axon in the remaining examples originated from a primary dendrite. Spinoparabrachial projection neuron axons showed variable trajectories either remaining within lamina I and projecting laterally (n = 10), projecting ventrally into deeper laminae (n = 4), projecting medially (n = 3), or extending rostrocaudally (n = 1). In addition, 10 recovered axons showed clear branching off the main parent axon giving rise to local collaterals (Figs. 7A, B). All axon terminals derived from SPBNs expressed immunolabelling for VGLUT2, and these were also closely apposed to Homer1-expressing puncta (Fig. 7C).

### 3.10. Heterogeneity within the lamina I spinoparabrachial projection neuron population

**Figure 8.**
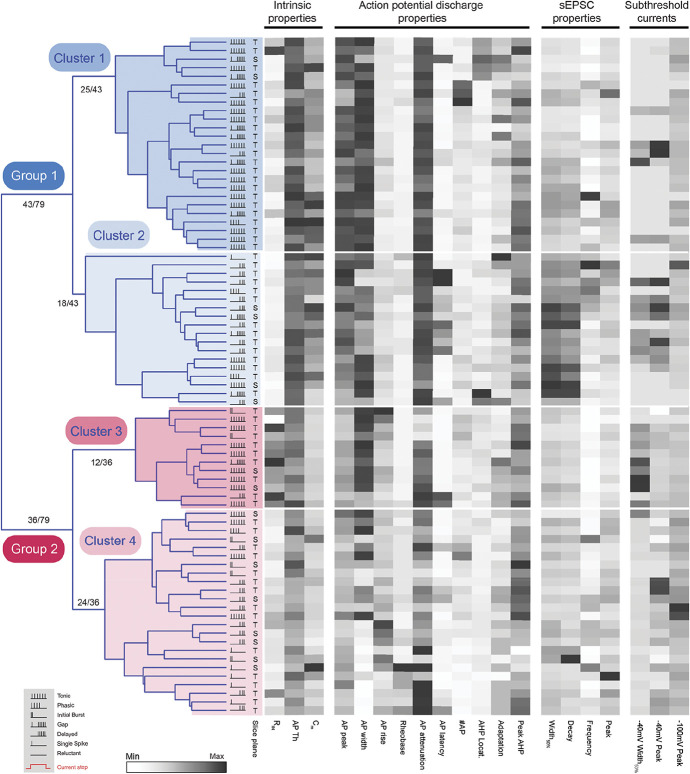
Hierarchical cluster analysis of LI SPBNs identifies distinct subpopulations. Dendrogram and heatmaps plotting the assignment of LI SPBNs into clusters based on their electrophysiological properties. The AP discharge patterns of each SPBN are annotated using the key in the lower left gray box (inset). Dendrograms show clusters (4 in this analysis) determined using a K-Means silhouette scoring method. Unsupervised clustering of SPBNs differentiated 2 major groups (blue and red) of approximately equal numbers (43 and 36), with each group subsequently further divided into 2 clusters (light and dark shading). Heatmaps present the relationship of 20 electrophysiological characteristics, normalised to maximum (black) and minimum (white) values for each property, aligned to each LI SPBNs cluster assignment. AP, action potential; SPBNs, spinoparabrachial projection neurons.

Given the heterogeneity in lamina I SPBN electrophysiological properties, we also undertook a hierarchical cluster analysis of this population in isolation, to distinguish potential SPBN groups/types. Morphological data were not included because only half the recorded LI SPBNs were adequately recovered and slice orientation varied. All other parameters (passive membrane, action potential, discharge pattern, subthreshold current, and sEPSC properties) were used to calculate Euclidian distances and construct dendrograms (Fig. 8). This analysis first identified 2 major LI SPBN groups of similar size (n = 43/79 and n = 36/79). Heatmaps comparing electrophysiological parameters showed that Group 1 LI SPBNs (blue group in Fig. 8) exhibited larger AP peaks, had more hyperpolarised AP thresholds, shorter AP discharge latencies, and tended to have lower input resistance and higher membrane capacitance (see Supplementary Table 2, available at http://links.lww.com/PAIN/B260). By contrast, Group 2 LI SPBNs (red group in Fig. 8) were less excitable with higher input resistances and more attenuated repetitive AP discharge (Supplementary Table 2, available at http://links.lww.com/PAIN/B260). The broad assignment into an easily excited and less excitable group of LI SPBNs was also supported by the incidence of AP discharge patterns. Group 1 LI SPBNs exhibited more rapid and longer discharge responses during current injection than Group 2 LI SPBNs (ie, TF, IB, PF, GF: 31/43, 70% vs 19/36, 53%, respectively). By contrast, those that required large and more sustained input to recruit AP discharge were more common in Group 2 (DF, SS, R: 13/43, 30% vs 17/36, 47%). Although not included in the analysis, the morphological characteristics of LI SPBNs reflected this group assignment (Supplementary Table 3, available at http://links.lww.com/PAIN/B260). For example, Group 1 LI SPBNs had larger soma, more extensive dendritic extensions in the rostrocaudal plane, and a smaller ventral dendritic spread consistent with lower membrane input resistance properties and larger capacitance values.

In addition to assigning LI SPBNs into 2 major groups, our hierarchical cluster analysis suggested that each group could be further subdivided into 2 clusters (Fig. 8). Along with the above properties that discriminated LI SPBN groups, a number of additional features including sEPSC kinetics, AP discharge, and individual AP characteristics helped distinguish these clusters (Supplementary Table 4, available at http://links.lww.com/PAIN/B260). Specifically, the more excitable AP discharge phenotypes were predominant in Clusters 1 and 3 (TF, IB, PF, GF: Cluster 1; 23/25, 92%; Cluster 3: 11/12, 92%). These 2 clusters could be separated, however, by other properties such as input resistance, AP threshold, AP peak, AP latency, and membrane capacitance. AP discharge patterns were more evenly distributed in Clusters 2 and 4 with the less excitable phenotypes also featuring (DF, SS, R: Cluster 2; 13/18, 72%; Cluster 4: 15/24, 63%). These clusters could be differentiated by sEPSC half-width, input resistance, AP peak, AP latency, AP threshold, sEPSC Tau, AP firing duration, −40 mV step response half-width, and rheobase current. Morphological characteristics of LI SPBNs clusters broadly reflected those of the SPBN groups outlined above but did not resolve cluster-specific features (Supplementary Table 5, available at http://links.lww.com/PAIN/B260). Together, these results support the general conclusion that LI SPBNs are not electrophysiologically homogeneous, rather they form a heterogeneous population paralleling the literature on dorsal horn interneurons.

### 3.11. Distinct features of the laminae III-V spinoparabrachial projection neuron population

**Figure 9.**
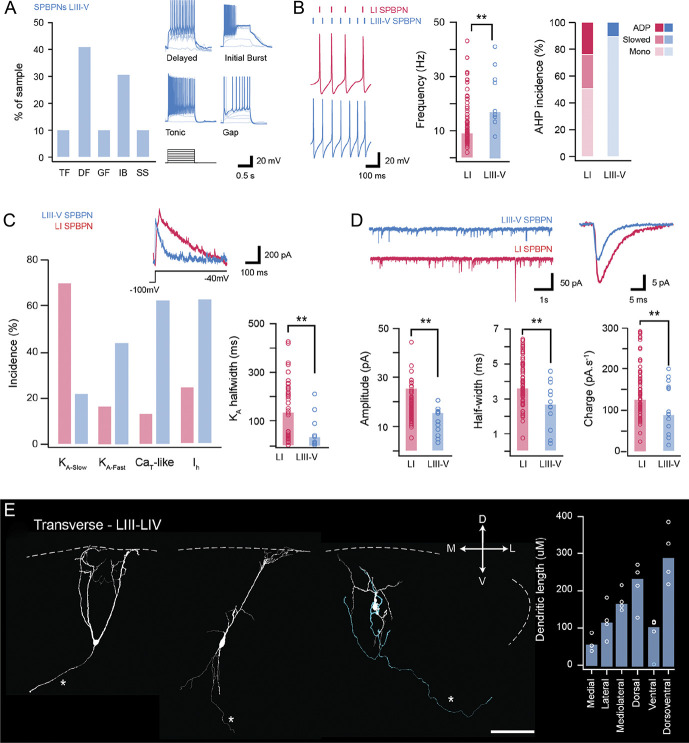
Properties of LIII-V SPBNs. (A) Plot shows the incidence of AP discharge patterns in LIII-V SPBNs. Discharge patterns included delayed firing (DF), initial bursting (IB), tonic firing (TF), gap firing (GF), and phasic (P). Delayed firing and initial bursting were most common in LIII-V SPBNs (see inset traces). (B) Traces compare AP discharge frequency in LI (upper red) and LIII-V SPBNs (lower blue). Dashes above highlight elevated spike frequency in the LIII-V populations. This is also clear in the group data plots of spike frequency (middle). Right plot compares the incidence of AHP profiles classified as having an afterdepolarization (ADP), slowed repolarisation (slow), or monophasic AHP (mono). Most AHPs exhibited monophasic profiles in LIII-V SPBNs. (C) Plot summarizes the incidence of subthreshold currents in LI and LIII-V SPBNs. Fast A-type potassium currents (K_A-Fast_) were the more prevalent in LIII-V SPBNs as were low-threshold T-type calcium currents (Ca_T_). Inset traces highlight the different decay profile of fast and slow A-type currents in LIII-V and LI SPBNs, respectively. Group data plot (lower right) compares A-type current half-width. Note, the faster decay of these currents in LIII-V SPBNs. (D) Traces (top left) show continuous sEPSC recordings from LIII-V (upper blue) and LI SPBNs (lower red). Overlaid traces (right) are average sEPSCs from associated recordings. Note: sEPSCs from LIII-V SPBNs exhibit lower amplitudes and slower decay kinetics. Group data plots (lower) compare sEPSC amplitude (left), half-width (middle), and charge (right). (E) Images show examples of LIII-V SPBN morphology recovered in transverse spinal cord slices after patch clamp recordings (dashed white line denotes the position of dorsal gray/white matter border). Red image shows a Neurobiotin-recovered LIII-V SPBPB in its maximum intensity z projection, with traced cell below (white). Several traced cells show the typical features of LIII-V SPBN morphologies, where their dendrites extend dorsally to reach the superficial dorsal horn. Group data plot (right) summarizes LIII-V SPBN dendritic dimensions in the rostrocaudal, mediolateral, and dorsoventral planes. Note: rostrocaudal measurements were not included because of tissue slice orientation. AHP, afterhyperpolarisation; AP, action potential; SPBNs, spinoparabrachial projection neurons.

Our viral injections also reliably labelled SPBNs in the deeper dorsal horn (LIII-V). This allowed us to characterise electrophysiological and morphological features in these projection neurons that have been overlooked by in vitro patch clamp studies. Some intrinsic membrane properties such as input resistance and resting membrane potential were similar in LIII-V and LI SPBNs (313 ± 40 MΩ vs 295 ± 22 MΩ, *P* = 0.75; −60.0 ± 3.0 mV vs −56.5 ± 1.0 mV mV, *P* = 0.27), whereas LIII-V SPBNs exhibited lower membrane capacitance (9.9 ± 0.9 pF vs 13.5 ± 0.5 pF, *P* = 0.006). Regarding action potential spiking, LIII-V SPBNs exhibited 5 patterns of discharge (Fig. 9A) including TF (20%), DF (33%), GF (10%), IB (20%), and single spike (10%). By comparison, TF was more prominent in LI SPBNs, whereas DF and IB were less common. AP discharge frequency also differed between SPBN populations with those in LIII-V exhibiting higher repetitive discharge frequencies than those in LI (22.4 ± 3.7 Hz vs 12.4 ± 1.0 Hz, *P* = 0.002, Fig. 9B). Consistent with this difference, subthreshold currents known to support repetitive discharge were more common in LIII-V SPBNs than those in LI (Ca_T_-like incidence of 62.5% vs 13.4%, *P* = 0.003; I_h_ incidence of 62.5% vs 25%, *P* = 0.039, Fig. 9C). By contrast, the incidence of A-type currents, which suppress AP discharge, was similar in LIII-V and LI SPBNs (62.5% vs 85.5%, *P* = 0.096). Other properties such as latency to AP discharge (97 ± 36 ms vs 126 ± 16 ms, *P* = 0.557) and AP discharge duration (633 ± 106 ms vs 665 ± 33 ms, *P* = 0.757) were also similar between LIII-V and LI SPBNs.

For the characteristics of individual action potentials, AP peak was smaller (47.4 ± 2.3 mV vs 55.3 ± 1.5 mV, *P* = 0.011) and AHP peak latency was shorter (9.21 ± 0.89 ms vs 15.59 ± 1.34 ms, *P* = 0.001) for LIII-V SPBNs compared with those in LI. Other AP characteristics such as AP threshold (−35.5 ± 1.8 mV vs −39.2 ± 0.7 mV, *P* = 0.062), AP rise time (2.67 ± 0.38 ms vs 2.17 ± 0.10 ms, *P* = 0.127), AP base width (5.34 ± 0.56 ms vs 5.62 ± 0.22 ms, *P* = 0.670), and AHP peak (−18.6 ± 1.7 mV vs −19.2 ± 0.6 mV, *P* = 0.710) were all similar in LIII-V and LI SPBNs. Some characteristics of excitatory input also differed in deeper and superficial SPBNs. For example, sEPSC amplitude was significantly smaller in LIII-V SPBNs compared with those within LI (11.9 ± 1.5 pA vs 18.4 ± 0.7 pA, *P* = 0.001, Fig. 9D). By contrast, the sEPSC frequency (4.5 ± 1.1 Hz vs 6.8 ± 0.7 Hz, *P* = 0.203), rise time (1.24 ± 0.10 ms vs 1.17 ± 0.03 ms, *P* = 0.482), and decay time constant (6.15 ± 0.57 vs 5.87 ± 0.24, *P* = 0.663) were all statistically similar. Smaller sEPSC amplitude did, however, contribute to a lower sEPSC charge in LIII-V SPBNs (97.6 ± 16.3 pA.ms vs 141.2 ± 6.9 pA.ms, *P* = 0.022) and reduced sEPSC excitatory drive (497.6 ± 144.5 vs 1077.0 ± 132.3 pA.ms.Hz, *P* = 0.005).

Finally, the morphology of a subset of LIII-V SPBNs was recovered from transverse slices (Fig. 9E, n = 4) allowing a comparison of somatodendritic dimensions. This showed that the mean soma size was similar in LIII-V and LI SPBNs (170 ± 28 μm^2^ vs 228 ± 23 μm^2^, *P* = 0.345); however, the mean dorsoventral dendritic length was greater in LIII-V SPBNs (292 ± 38 μm vs 149 ± 13 μm, *P* = 0.001), but the mean mediolateral dendritic length was smaller (172 ± 15 μm vs 263 ± 13 μm, *P* = 0.001). These dimensions reinforce the striking appearance of LIII-V SPBNs, often with long dendritic extensions spanning dorsally into the superficial dorsal horn laminae. Among these reconstructions, one LIII-LV SPBN exhibited dendrite protruding into the dorsal white matter. In addition, an axon was identified in all LIII-LV SPBNs, with 2 examples showing a medial trajectory towards the central canal, and the remaining taking a course towards the lateral funiculus of the ipsilateral dorsal horn. One of these axons was also observed to give rise to axon collaterals that extended dorsally, reaching ∼LIII/LII before being truncated.

## 4. Discussion

This study provides a comprehensive characterisation of spinal projection neurons that relay information to the PBN in the mouse. We find that SPBNs in the mouse exhibited a range of electrophysiological properties that differentiated them from neighbouring unlabelled neurons. Using unbiased hierarchical cluster analysis of electrophysiological properties, we found that SPBNs in lamina I can be differentiated into 4 functionally distinct subpopulations. Spinoparabrachial projection neurons located in laminae III-V exhibited electrophysiological and morphological features that distinguished them from lamina I SPBNs, including higher AP discharge frequencies and weaker excitatory inputs. Approximately 80% of SPBNs in lamina I, and approximately half of those in laminae III-IV, showed immunolabelling for the NK1 receptor. Axon from both populations of SPBNs gave rise to local collaterals, and we provide anatomical evidence that these branches are a source of excitatory input to local circuits. The role of SPBNs in transmitting nociceptive signals in both acute and chronic pain settings is well established,^14^ and the data presented here help establish the contribution of these projection neurons to spinal pain processing through their likely recruitment of (as yet) unidentified dorsal horn circuits.

In the context of these results, some technical considerations for collecting a large sample of multiple active and passive neuronal properties should be noted. For example, a pharmacological approach to unambiguously identify ionic currents (synaptic or intrinsic) would preclude complete characterisation in each recording. Thus, we rely on voltage threshold relationships along with temporal current/voltage features to distinguish electrophysiological characteristics and achieve the large data set required for our cluster analyses. Future work will be required to clarify the precise nature of channels and receptors underlying some electrophysiological differences we report. Likewise, analysis of neuronal morphology in slice preparations represents a compromise. Specifically, slice preparation likely prevents full assessment of neuronal morphology; however, this approach does allow widespread targeted recordings of labelled populations across the dorsal horn as well as in deeper laminae. By contrast, intact or ex vivo preparations that fully preserve neuronal morphology tend to be limited to younger animals where adult levels of myelination are incomplete. The intact approach also typically biases sampling to more superficial and lateral DH territories where myelin is thinnest, improving visualisation for targeted recording. We address the shortcomings of neuronal recovery from slices by reporting morphological data from 2 different slicing planes but acknowledge our values may still underpredict the full extent of mouse SPBNs. Regardless, we confirm extensive dendritic territories and axonal branching that extend in multiple planes.

Viral labelling of PNs also introduces some experimental caveats when compared with studies that have typically used a lipophilic tracer (DiI) or *post hoc* analysis of axonal trajectory to establish PN identity. Specifically, different tracer uptake, transport, and end labelling mechanisms may influence the populations identified. We purposely sought to maximally label PNs by targeting the PBN, which is the major ascending target for rodent PNs.^15,42^ Furthermore, previous work has demonstrated substantial collateralisation of PN axon terminations, with axon collateral terminals in the PBN, PAG,^3^ inferior olivary complex, and thalamus.^15^ Labelling considerations may have also impacted the cells we termed “unlabelled” neurons. We cannot exclude the possibility that a fraction of PNs, not labelled by brainstem injections, are included in these unlabelled recordings. For example, slow A-type currents, previously associated with SPBNs in rat,^44^ were identified in some UN recordings. Despite this, several significant differences argue LI SPBNs and UNs do differ, and thus the UN sample is enriched with local LI interneurons.

Several studies have reported electrophysiological properties from putative and identified PNs in rodents. For example, putative PNs identified by a relatively thick axon running ventromedially toward the contralateral spinal cord have been studied in hamster.^24^ The input resistance of this PN sample was lower than neighbouring laminae I-II cells, and the rostrocaudal extent of PN dendritic arborisations was substantially larger for other cell types. These observations were largely replicated in rat PNs,^44^ identified by retrograde labelling from PBN and PAG. Mouse LI SPBNs had a similar input resistance to unidentified neurons, although membrane capacitance was higher in the SPBN population. This may represent a species difference; however, PN studies commonly target large neurons because the literature associates large soma size with PNs.^2^ We recorded from cells based on mCherry expression alone, without considering soma size. A close inspection of LI SPBN input resistance across our sample (Fig. 8) reinforces this point, as it includes a population of SPBNs with high input resistance (Cluster 3) as has other recent work describing a cold selective population of projection neuron.^26^

Electrophysiologically, 2 novel forms of AP discharge have been described in rat PNs that project to the PBN or PAG, respectively.^44^ Specifically, the gap firing pattern that was mediated by a slow A-type potassium current dominated in parabrachial PNs, whereas burst firing, produced by a low-threshold calcium current, was distinctly expressed in PAG PNs. Importantly, these discharge patterns were only present when cells were activated at very hyperpolarised membrane potentials (∼−80 mV). Furthermore, recent work suggests the gap discharge patterns may not be unique to PNs as they have also been described in LI interneurons; however, bursting patterns remain closely associated to PNs.^38^ By contrast, gap and burst firing were not reported in putative hamster PNs, although discharge was assessed from −60 mV in this work.^24^ In mouse, we assessed AP discharge from a membrane potential of −70 mV, which also could have obscured gap or burst firing. Nevertheless, our LI SPBN sample contained gap firing responses and our voltage clamp analysis showed 60% of mouse LI SPBNs expressed slow A-type potassium currents (implicated in gap firing), and some (∼10%) also exhibited low-threshold calcium-like currents (associated with burst firing). Mouse LI SPBNs also exhibited more hyperpolarised AP thresholds, along with slowed AP repolarisation phases, or afterdepolarisations. Similar AP characteristics have also been reported for rat projection neurons.^44^ Thus, our data on AP discharge suggest that many electrical features observed in rat are conserved in mouse PNs.

The proportions of discharge patterns within our UN sample vary from other studies of LI interneurons. We describe most UNs as having a delayed discharge phenotype, closely mirroring proportions that we have reported in LII, with tonic, IB patterns, and reluctant patterns also identified. This distribution contrasts recent work in rat that showed most LI interneurons exhibited TF, along with some IB and delayed discharge responses.^19^ This work also highlighted that a significant proportion of these interneurons exhibited plateau potentials shown to be dependent on L-type calcium channels. By contrast, we did not observe evidence of plateau potentials from our UN data. These differences could be due to the baseline membrane potential (our data: −70 mV vs resting membrane potential), differences in cell targeting (across the mediolateral extent vs lateral only), species, and developmental age of the animals (P7-P12 vs P28-P56).

In addition to providing a detailed characterisation of mouse LI SPBNs, our data show these cells can be split into 4 clusters. Such subdivision of PNs is not new, with previous studies segregating this population on neurochemistry,^43,49^ morphology,^35^ modality specific responses,^16,29^ projection targets,^44^ and function.^15^ The difference here is that we have taken an unsupervised approach and used electrophysiological properties to define populations with similar characteristics. We have also recently used this approach in genetically identified inhibitory and excitatory dorsal horn interneurons.^9^ Interneurons clustered to form 2 pure excitatory groups and 3 high-purity inhibitory groups. The data presented here suggest LI SPBNs can also be reliably segregated into groups in this way.

The existence of distinct LI SPBN clusters, with different electrophysiological characteristics, suggests clusters process information differently. This aligns with recent work in rat describing 3 functionally distinct PN groups, distinguishable on AP spiking evoked by dorsal root (afferent) input.^1^ High-output PNs responded with extended AP discharge, low-output PNs responded with a single AP, and a small proportion of PNs exhibited intermediate responses. Among other features, burst firing during depolarising current injections was prominent in the high-output subpopulation. This suggests an association between low-threshold T-type calcium currents, which mediate burst firing,^32^ and high-output PNs. Our Cluster 4 LI SPBNs express transient inward currents with properties matching T-type currents (5/6 LI SPBNs), suggesting correspondence with the high-output rat PN category.

Sensory modality responsiveness has also been used to classify PNs, with a recent study showing most mouse LI SPBNs (∼85%) are nociceptive, many exhibit polymodal responses, and approximately a third are preferentially tuned to a single modality (often cold).^4^ As these experiments used extracellular recording, information on intrinsic and cellular properties is not available. Fortunately, work using a semiintact ex vivo mouse preparation, including the spinal cord with skin attached, bridges this knowledge gap.^25^ Experiments using this preparation have shown that cold sensing SPBNs have small somas, high input resistance, and low-frequency spontaneous excitatory synaptic inputs.^26^ Collectively, these properties are shared by our Cluster 3 and suggest cold-specific SPBNs correspond with this cluster.

Most recently, work using a suite of genetic and behaviour paradigms, along with the skin attached preparation described above, has demonstrated distinct SPBNs relaying nociceptive and affective touch information to the brain.^15^ Specifically, TACR1-positive SPBNs had higher mechanical and thermal thresholds compared with the GPR83-positive SPBNs, as well as decreased AP firing frequency with peripheral stimulation. Furthermore, TACR1 PNs responded to nociceptive-specific afferent input, whereas the GPR83 line did not, instead encoding a wider range of intensities including affective touch. As this study did not report detailed membrane properties, it is difficult to align the TACR1 and GPR83 populations with the clusters we describe. It is, however, noteworthy that GPR83 SPBNs generally exhibited higher instantaneous firing frequencies during peripheral stimulation than TACR1 SPBNs. These features suggest correspondence with our Group 1 SPBNs, which exhibited easily recruited to sustained repetitive AP discharge, and Group 2 SPBNs with more difficult to recruit phenotypes, respectively.

Our study also provides information on the intrinsic properties and AP discharge of deep SPBNs within laminae III-V. These cells represent a much smaller population, with work in mouse reporting only ∼9 SPBNs in LIII-V per dorsal horn in the L4 segment (vs 238 SPBNs in LI),^11^ mirroring previous work in rat.^3^ Although information on the membrane properties of deep SPBNs is lacking, these cells overlap with PNs targeting the thalamus that have been studied in monkey, cat, and rat^50^ and typically exhibit wide-dynamic-range (WDR) responses to low-threshold light touch and high-threshold nociceptive stimuli. The morphology of mouse LIII-V in our study is consistent with features of these WDR neurons (Fig. 9) and is also reminiscent of LIII antenna interneurons, shown to exhibit WDR responses. Despite appearing morphologically similar, rat LIII antenna interneurons exhibited tonic, or rhythmic spontaneous discharge in Ref. 20, whereas DF and IB were prominent in our LIII-V SPBN sample. Mouse LIII-V SPBNs also fired APs at almost twice the rate of LI SPBN neurons, regardless of discharge patterns, and received weaker excitatory drive that may relate to inputs on peripheral dendrites undergoing greater electrotonic filtering. Together, these observations suggest that although both populations relay sensory information to the lateral PBN, their modality content and signal coding features differ.

Morphological analysis of lamina I SPBNs showed that dendrites from these cells were largely contained within lamina I, but that many cells displayed ventrally directed dendrites that extend into laminae II and III. Similar morphologies have been described in both TACR1-expressing and GPR83-expressing SPBNs, respectively,^15^ and these provide an anatomical framework through which low-threshold mechanosensory input in deeper dorsal horn laminae can recruit pain circuits under pathological conditions. Finally, our anatomical data establish that SPBNs in the mouse give rise to local axon collaterals, and these observations support previous findings in monkey, cat, and rat.^5,6,47^ Axon collaterals from lamina I projection neurons in young rats have been defined on the basis of their location and the extent of collateral spread,^47^ but the incomplete recovery of axons in our slice preparations prevented us from assigning mouse SPBN axon collaterals to these categories. Nonetheless, our data expand on these earlier findings by demonstrating that axon terminals from these collaterals express VGLUT2 and appose Homer1-immunoreactive puncta. These observations provide anatomical evidence that collaterals derived from SPBNs are a source of excitatory synaptic input to (as yet) unidentified dorsal horn circuits.

In summary, our experiments provide new and detailed information on the functional properties of mouse SPBNs. We show that many LI SPBN membrane properties reported in other rodent species are conserved in mouse. Further analysis differentiated 4 electrophysiologically distinct clusters of mouse LI SPBNs. This suggests functionally discrete LI SPBN subpopulations exist to play different roles in relaying sensory signals to the brain as has been recently highlighted.^15^ Examples of correspondence between the LI SPBN clusters and other classifications based on afferent responsiveness^1^ and modality selectivity^26^ reinforce the heterogeneity of these important spinal cord output neurons. We also provide the first data on intrinsic and synaptic properties of deeper LIII-V SPBNs and show they are electrophysiologically distinct from their more superficial counterparts in lamina I. These deep SPBNs can now be added to our increasingly detailed view of dorsal horn circuits. Finally, we provide evidence for axon collaterals from SPBNs forming excitatory synapses within the DH in mouse, reinforcing an important but underappreciated role for projection neurons in spinal sensory processing. This baseline information will assist future studies on the role of SPBNs in pathological pain and help our search for molecular targets that restore dorsal horn circuit function.

## Conflict of interest statement

The authors have no conflicts of interest to declare.

## Appendix A. Supplemental digital content

Supplemental digital content associated with this article can be found online at http://links.lww.com/PAIN/B260.

## Supplemental video content

A video abstract associated with this article can be found at http://links.lww.com/PAIN/B261.
